# Vitamin D Status and Its Associated Risk Factors among Adults in the Southwest Region of Cameroon

**DOI:** 10.1155/2018/4742574

**Published:** 2018-03-19

**Authors:** Delphine A. Tangoh, Tobias O. Apinjoh, Yasir Mahmood, Robert V. Nyingchu, Beatrice A. Tangunyi, Emmanuel N. Nji, Abid Azhar, Eric A. Achidi

**Affiliations:** ^1^Faculty of Health Sciences, University of Buea, Buea, Cameroon; ^2^Faculty of Science, University of Buea, Buea, Cameroon; ^3^Dr. A. Q. Khan Institute of Biotechnology and Genetic Engineering (KIBGE), University of Karachi, Karachi, Pakistan

## Abstract

**Background:**

Vitamin D has been shown to exert its actions on the musculoskeletal, gastrointestinal, prostate, renal, endocrine, immune, and cardiovascular systems. Current reported data of hypovitaminosis D reveals a global pandemic, with an estimated one billion people worldwide presenting with hypovitaminosis D.

**Objective:**

This study aimed at investigating the vitamin D status and its associated risk factors in Cameroonians from the South West Region.

**Method:**

The study was a community- and hospital-based prospective longitudinal study. It was carried out during the dry and rainy seasons between the months of July and December 2015 in the South West Region of Cameroon involving 372 participants aged 35 years and above. After obtaining informed consent, a structured questionnaire was used to capture demographic data and risk factors of vitamin D deficiency. Blood samples were collected from the volunteer participants in the peak months of the rainy season and dry season, and the serum used to analyse for vitamin D by ELISA and calcium by spectrophotometry. 25(OH)D levels ≥75 nmol/L (≥30 ng/mL) were considered sufficient while levels <75 nmol/L were considered as hypovitaminosis D (insufficiency/deficiency).

**Results:**

Hypovitaminosis D (deficiency/insufficiency) was prevalent in 25.8% (96) of the study population, with only 3.2% (12) deficiency and 22.6% (84) insufficiency. There was a significant inverse relationship (*r*=−0.119, *p*=0.02) between age and 25(OH)D levels; however, this relationship was not significant when controlled for gender, number of hours spent outdoors, and percentage of body covered. Gender, ethnic origin, percentage of body covered, time spent outdoors, and season did not influence serum vitamin D levels.

**Conclusion:**

Results of this study suggest that the prevalence of hypovitaminosis D is relatively low in this study population and only age is a risk factor of vitamin D deficiency.

## 1. Introduction

The vitamin D content of the human body is synthesized from the precursor molecule 7-dehydrocholesterol (7-DHC) in the skin by the action of ultraviolet B (UVB) irradiation (280–320 nm) from sunlight. The UVB radiation converts 7-dehydrocholesterol in the skin to vitamin D3 which is then transported to the liver for hydroxylation to 25-hydroxyvitamin D [25(OH)D]. 25(OH)D is then transported to the kidney where another hydroxylation occurs forming 1,25-dihydroxyvitamin D (1,25(OH)_2_D_3_) which is the active form of vitamin D, referred to as vitamin D hormone [1, 2].

About 90% or more of the circulating vitamin D in the body is contributed by cutaneous production, while the remaining portion comes from diet. Though 25(OH)D is not the active form of the hormone, its measured blood level is accepted for the determination of vitamin D status because it is the most stable form of vitamin D metabolites and has a longer blood half-life [3].

There is growing evidence in literature of the diverse function of the vitamin D hormone (1,25(OH)_2_D_3_). Primarily, vitamin D induces the proteins involved in active intestinal calcium absorption and also stimulates active intestinal absorption of phosphate. It also has the ability to mobilize calcium from the bones in the absence of dietary calcium [2]. A growing world of knowledge is being produced revealing the noncalcaemic actions of vitamin D due to the wide distribution of the vitamin D receptor. Vitamin D has been shown to exert its actions on the musculoskeletal, gastrointestinal, prostate, renal, endocrine, immune, and cardiovascular systems [4].

The earliest discovery of vitamin D deficiency was rickets in children and osteomalacia in adults. A lot of recent studies have revealed that low vitamin D levels are associated with conditions such as osteoporosis, cancer, diabetes mellitus, metabolic disease, cardiovascular disease, infections like tuberculosis, and autoimmune diseases such as multiple sclerosis. There is still a large research gap to relate causality of vitamin D deficiency for most of the outcomes [5].

Currently, there is no worldwide agreement on the optimal levels of serum 25(OH)D. The Institute of Medicine (IOM) in 2011 recommended serum 25(OH)D level ≥50 nmol/L as adequate because it covered the requirements of at least 97.5% of the population reviewed [6]. Nevertheless, vitamin D deficiency is defined by most experts as 25(OH)D levels of <50 nmol/L (<20 ng/mL). Levels between 50 and 75 nmol/L (20–30 ng/mL) indicate “insufficiency,” and a level ≥75 nmol/L (≥30 ng/mL) indicates “sufficiency” [7, 8]. Based on these cutoffs, the current reported data of hypovitaminosis D reveals a global pandemic, and it has been estimated that about one billion people, worldwide, have 25(OH)D deficiency or insufficiency [9]. The prevalence of serum 25(OH)D deficiency/insufficiency reportedly varies between 30% and 93% [8, 9]. The highest prevalence of hypovitaminosis D has been reported in temperate climate regions that receive limited sunlight, especially during winter [10]. Vitamin D deficiency/insufficiency is also prevalent in the Middle East because of their religious and cultural demand of covering most of the body parts [11]. Also, recent studies have revealed that vitamin D insufficiency is common in tropical countries, such as Malaysia and Vietnam [12, 13]. Studies in the United States have demonstrated significant discrepancy in vitamin D status among Caucasian, African American, and Hispanic men, highlighting the influence of race on vitamin D status [14]. A study among elderly people in some villages in the Southwest region of Cameroon in 2002 revealed that 24% of the participants presented with vitamin D levels below normal range [15]. Some of the associated risk factors of vitamin D deficiency were age, race, body mass index, use of medications known to affect vitamin D metabolism, lack of exercise, inadequate amount of vitamin D in food, and low sun exposure. In a study in East Africa where there is equally sufficient sunshine and maximum outdoor activities, the prevalence of vitamin D deficiency was relatively low [16], reflecting the effect of sunlight and outdoor exposure to vitamin D status. The prevalence of vitamin D insufficiency has been revealed to range from 43% in subjects with one risk factor to 90% in those with five or more risk factors in many studies [17].

There is still limited data with respect to vitamin D status in tropical Africa and relatively no current data in Cameroon which is situated near the equator with sufficient sunshine. Thus, the main objective of this study was to investigate vitamin D status and its associated risk factors in residents from the Southwest region of Cameroon. The results may provide useful data that can influence policies to improve on the overall health status of the community.

## 2. Methodology

### 2.1. Study Design and Study Population

The study was a community- and hospital-based prospective longitudinal study carried out during the dry and rainy seasons between the months of July and December 2015 in the Southwest region of Cameroon. A total of 372 consenting nonpregnant participants aged 35 years and above were enrolled by convenient sampling into the study. Most of the participants were recruited from three communities representing rural, semirural, and urban settings within the Fako division. The rest of the participants were those who visited Mount Mary Hospital in Buea and St. Francis Polyclinic in Kumba for CVD screening or for CVD follow-up treatment.

### 2.2. Study Area

Southwest region is one of the ten regions in Cameroon, and the indigenes are mainly the Bantus ethnic group, with many Semi-Bantus as settlers and dark-skinned colour (type 5 and 6). The area is characterised by a forested equatorial climate, comprising two seasons; a short dry season (November–March) and a long rainy season (March–November), and ambient temperatures vary from 18°C in August to 35°C in March [18].

The communities included Ombe native for rural setting, comprised of mostly farmers; Bolifamba for semirural setting, comprised of a mixture of farmers, peti traders, and people with blue-collar jobs; Molyko and its environs for urban setting, made up mainly of residents with white-collar jobs and business people including students of tertiary institutions. The main staple foods in this region include tubers (cocoyams and cassava), plantains, a variety of green leafy vegetables, and fresh or smoked fish as source of proteins [19].

### 2.3. Ethical Considerations

Ethical clearance was obtained from the Faculty of Health Sciences Institutional Review Board of the University of Buea. Administrative clearance was obtained from the Regional Delegate of Public health in the Southwest region and the Directors of the participating hospitals. Furthermore, authorization was obtained from the chiefs and quarter heads of the communities (Ombe native, Bolifamba, and Molyko) that participated in the community-based study, while the heads of the health establishments involved in the hospital-based study granted authorization. Also a written informed consent was obtained from those who voluntarily accepted to participate in the study after adequate sensitization of the study objectives and protocol. A code was provided to the participant, and their names and contact addresses were confidentially kept by the principal investigator only.

### 2.4. Subject Recruitment

The study subjects were recruited in the heart of the rainy season (July and August). Two hundred and sixty-four (264) participants were recruited from three communities representing rural (45), semirural (93), and urban (126) settings. One hundred and eight (108) participants were also recruited from two hospitals (Mount Mary Hospital in Buea and St. Francis Polyclinic in Kumba) where a team of specialists consult and follow-up patients at risk of CVD or with a CVD. For the community-based recruitment, announcements were sent to churches within these communities to inform the people of the study, recruitment requirements, and assembly venue. The participants voluntarily assembled at designated focal points within the selected communities between 7 : 30 and 12 : 00 am after an overnight fast each sampling day. After adequate sensitization by the unit heads, informed consent form with information on project objectives and protocol was given to every participant for appraisal and signature. For the hospital-based study, people who visited these hospitals for a CVD screening or follow-up of an existing CVD were informed of the study and recruited upon consent. Volunteers were assisted to complete a structured questionnaire (see Supplementary Materials (available here)) to obtain demographic data and other information with relation to the risk factors of vitamin D deficiency.

The participants were invited for a second round of sample collection in December (after 4 months), representing dry season. Only 16.4% (61/372) of the participants made it for this second round of sample collection.

### 2.5. Blood Collection and Analysis

The first blood samples were collected from the study participants in the peak months of the rainy season (July–September). Blood samples (4 ml) were collected from the participants by venepuncture into a dry tube (4 ml) using a 5 ml nontoxic, pyrogen-free, sterilized disposable syringes (Cathy Yougo®, France). The blood samples were then centrifuged (BECKMAN Instruments Inc., USA) at 2500 rpm for 5 minutes, and serum was transferred into separate eppendorf tubes using sterile micropipettes and stored in the freezer at −35°C for subsequent batch analysis of serum 25(OH)D and calcium levels. A second sample (4 ml) was collected from participants in the dry season (December) who accepted to come for a repeat of the above measurements in order to assess the influence of seasonal variation on vitamin D status.

Serum total calcium levels were measured together with quality control material (Cypress Diagnostics, Belgium Normal Control Human Serum, code: HBC01) by colorimetry using a commercial reagent kit (Calcium OCC; Linear Chemicals S.L., Spain). Following the manufacturer's procedure, 10 *μ*L of serum/standard/quality control material was mixed with 1 ml of working reagent and incubated at room temperature for 2 minutes, and the absorbance was measured at 570 nM with a spectrophotometer (Jenway Ltd, UK). The concentration of serum calcium was calculated using the absorbance and concentration of the standard. The reference range considered as normal total calcium was 8.4–10.2 mg/dL (2.1–2.5 mmol/L).

Measurement of serum 25(OH)D was done by a competitive ELISA technique with a selected monoclonal antibody recognizing 25(OH)D using a commercial kit called 25(OH)D Xpress ELISA kit (Immundiagnostik AG, Germany) and by following the manufacturer's protocol. The reagent kit was reported to have a specificity of 100% for 25(OH)D_3_ and 67% for 25(OH)D_2_ with a linearity of 6.4–250 nmol/L and a high correlation (*R* ^2^ = 0.92) with LC-MS/MS reference standard method. 10 *μ*l of serum sample/calibrator/control A and B was mixed with 300 *μ*l of a patented release agent and was used to release 25(OH)D from the 25(OH)D-VDBP-complex, votexed, and 100 *μ*L was transferred into 25(OH)D precoated wells on an ELISA microtiter plate and incubated. Later, an anti-25(OH)D antibody was added, and the 25(OH)D in the sample and a fixed amount of 25(OH)D bound to the microtiter well competed for the binding of the antibody. Then, a peroxidase-conjugated antibody was added into each microplate well. Finally, an acidic stop solution was added to terminate the reaction, whereby the colour changes from blue to yellow. The intensity of the yellow colour was inversely proportional to the 25(OH)D concentration of the sample. Samples were quantified by referring their optical density to a lot-dependent master calibration curve and the use of the calibrator that was analysed with each test. Results were considered valid only when the values of the control A and B were within the manufacturer's recommended range. Concentrations of 25(OH)D < 50 nmol/L (<20 ng/mL) were considered as deficient, while values 50–74 nmol/L (20–29 ng/mL) were considered as insufficient, and values ≥75 nmol/L (≥30 ng/mL) were considered sufficient (adequately supplied).

### 2.6. Data Analysis

Data was analysed using the statistical software package IBM SPSS statistics for windows, version 20 (SPSS Inc., USA). The data collected upon first recruitment of participant were used for the statistical analysis, while the second data collected from participants on second visit were used only for evaluation of seasonal variation. Normality of the continuous data was evaluated using Kolmogorov-Smirnov test. Data were expressed as percentages, mean ± standard errors of the mean (SEM) for normally distributed continuous data and median with interquartile range (IQR) for nonnormal continuous data. The chi-squared test was used to measure the difference in frequency for categorical variables. The Student's *t*-test or Analysis of Variance (ANOVA) was used to assess the difference between group means of parametric variables, while Mann-Whitney and Kruskal-Wallis tests were used to compare groups of independent nonparametric variables. Wilcoxon test was used to compare paired nonparametric variables such as 25(OH)D levels in rainy and dry season. Pearson's correlation coefficient and Spearman's rank correlation coefficient were used to evaluate the relationship between continuous parametric and nonparametric variables, respectively. Statistical significance was designated as *p* ≤ 0.05.

## 3. Results

### 3.1. General Characteristics of the Study Population

A total of 372 volunteers were enrolled into the study with 112 (30.1%) being males and 260 (69.9%) females. The mean (±SEM) age (years) was 53.7 ± 0.6, ranging from 35 to 85 years and the most represented age group was 50–59 years (30.4%). There was no significant difference (*p*=0.30) in the mean age between males (52.8 ± 1.2 years) and females (54.1 ± 0.7 years). The ethnic origin of the participants consisted mostly of the Semi-Bantus (228, 62.6%) followed by the Bantus (136, 37.4%). Majority of the participants were married (57.1%), while those who were widowed accounted for 27.5% of the study population. Participants with primary level of education constituted the highest percentage (34.1%), while those with tertiary education were least represented (20.8%). Most of the participants (58.8%) had a monthly income of less than 100,000 frs CFA, 15.1% had income greater than 200,000 frs CFA, while 14.6% had no form of income, and only 15.3% (57) of the participants owned cars. For the community-based study, 126 (47.7%), 93 (35.2%), and 45 (17.1%) participants were recruited from the urban, semirural, and rural community settings, respectively. Only fourteen (3.8%) of the study participants smoked any type of cigarettes, of these 7 (1.9%) smoked regularly and the remaining 7 (1.9%) smoked occasionally.

### 3.2. Vitamin D Status and Potential Risk Factors

**Figure 1:**
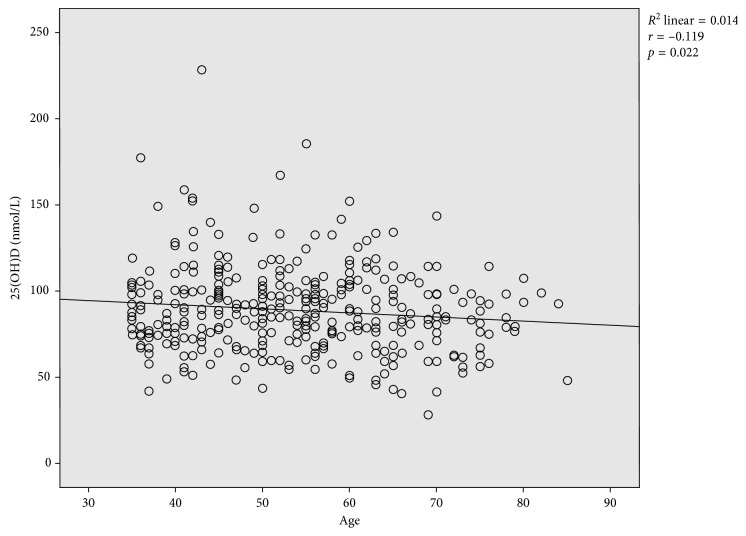
Correlation of serum 25(OH)D (vitamin D) levels with age in the study population.

The median (IQR) level of 25(OH)D (vitamin D) in the study was 87.2 (26.6) nmol/L with minimum value of 28.9 nmol/L and maximum value of 288.5 nmol/L. No significant difference (*p*=0.22) of median 25(OH)D levels were observed between males (89.8 nmol/L) and females (86.0 nmol/L). There was a significant inverse relationship (*r*=−0.119, *p*=0.02) between age and vitamin D levels, with vitamin D levels generally decreasing with increasing age (Figure 1). The correlation of vitamin D levels with age was not significant (*p*=0.37) when controlled for gender, number of hours spent outdoors, and percentage of body covered. There was no statistically significant difference in the median vitamin D levels between age groups (*p*=0.06) (Table 1).

Median vitamin D levels were not significantly different (*p*=0.25) between the Semi-Bantu (88.8 nmol/L) and the Bantu (84.9 nmol/L) ethnic groups. Furthermore, no statistically significant (*p*=0.12) association was observed between median 25(OH)D levels and the type of community (Table 1). There was no significant difference of median vitamin D level between levels of education (*p*=0.54) and income levels (*p*=0.09). Marital statuses, average number of hours spent outdoors, and percentage of the body covered did not have any significant effect on the median vitamin D levels. Also, rainy or dry seasons had no significant effect (*p*=0.64) in the median vitamin D levels (Table 1).

With regard to vitamin D deficiency, 25.8% (96) of the study population had hypovitaminosis D (<75 nmol/L), which included 3.2% (12) with deficiency (<50 nmol/L) and 22.6% (84) with insufficiency (50–75 nmol/L). As presented in Table 2, there was no significant association in vitamin D status (hypovitaminosis D versus sufficiency) with gender (*p*=0.70). There was also no significant difference (*p*=0.12) in vitamin D status between the Bantu and the Semi-Bantus ethnic groups (Table 2). There was no statistical difference in vitamin D status between the level of education (*p*=0.89), income level (*p*=0.72), and marital status (*p*=0.78). No significant difference (*p*=0.21) was observed in vitamin D status with respect to time spent outdoors. There was also no statistical difference (*p*=0.06) of vitamin D status and percentage of body covered (Table 2).

**Figure 2:**
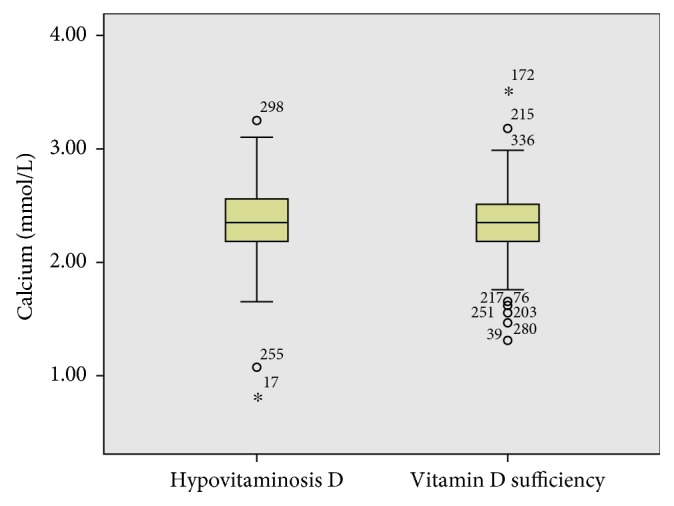
Calcium distribution between hypovitaminosis D and vitamin D sufficiency.

Only 47 (12.7%) of the study participants had low levels of total serum calcium, while 241 (65.1%) had normal levels and 82 (22.2%) had high calcium levels. There was no significant correlation between vitamin D levels with calcium levels (*r*=0.043, *p*=0.41). In addition, there was no significant difference in calcium levels between those with sufficient vitamin D and hypovitaminosis D (*p*=0.72) (Figure 2).

## 4. Discussion

The overall prevalence of vitamin D levels below 75 nmol/L (hypovitaminosis D) in this study was 25.8% and only 3.2% presented with overt deficiency (<50 nmol/L; <20 ng/mL). This finding is similar to results previously obtained among elderly people in some villages in Kumba health district in 2002 where 24% of the study participants presented with hypovitaminosis D [15]. Results similar to our finding were reported in a study in Malaysia involving Chinese and Malaysian men aged 20 and above with hypovitaminosis D of 23.2% [5]. The similarity with our study could be as a result that they included a wider range of age groups as this study, and also our dressing style is similar to the Chinese. Also a study of traditionally living populations in East Africa with expected maximum sun exposure due to extensive outdoor activities with light dressing revealed no vitamin D deficiency and only 13.3% vitamin D insufficiency [20], confirming that lifestyle greatly influences vitamin D status.

The prevalence of hypovitaminosis D was relatively very low in this study when compared with studies in the Middle East where there is equally sufficient sunshine. The study of Al-Horani et al. [21] among Iraqis and Jordanians revealed hypovitaminosis D of 67.4% and 92.2%, respectively. The marked difference observed could be partly as a result of differences in lifestyle, especially dressing style amongst others. Also high prevalence of hypovitaminosis D of 66.7% was observed in a study carried out in Brazil among elderly men of pigmented skin colour in the peak of the sunny season [22]. The results of this Brazilian are different from our findings probably because they used only elderly participants, while we recruited participants within a wide age range. An overall pooled estimate of results from many European countries, irrespective of age group, ethnic mix, and latitude of study populations, showed a vitamin D deficiency (<50 nmol/L) of 40.4% [23], which is far much higher than what was observed in our study. The National Health and Nutrition Examination Survey (2005–2006) of adults in the USA identified an overall vitamin D deficiency prevalence rate of 41.6%, with the highest rate seen in blacks (82.1%) [14]. All of these observations may probably reflect the impact of lifestyle and environmental factors in vitamin D status.

This study revealed no significant difference of vitamin D status between females and males This is in conformity with results of a systematic review of vitamin D status in populations worldwide which did not reveal any substantial gender-related differences in vitamin D status, except in the Middle East and Asia, probably due to the dressing code of females in these regions [24, 25]. Contrarily, a study in Lebanon revealed that females had significantly higher vitamin D levels than males [26].

Vitamin D levels significantly decreased with increasing age. This is expected, since the skin thickness decreases with age and thus, the production of 7-DHC is also compromised. Also, it could be due to the fact that with advancing age, a more sedentary and indoor lifestyle is easily adopted. The result of this study is in accordance with that obtained in the USA and Central Europe which observed reduced levels of vitamin D with older participants [27, 28]. They also proposed that the decrease in 25(OH)D with respect to age was attributed to decreased time spent in the sun and decreased vitamin D production efficiency. Contrary to these findings, a study in East Africa found a positive relationship of vitamin D and age [16]. This could be expected since their population did not include ages above 64 years. However, the relationship of vitamin D levels with age was not significant when controlled for gender, number of hours spent outdoors, and percentage of body covered. This observation reflects the probable significant impact of lifestyle and environmental factors in vitamin D status as mentioned earlier.

There was no significant difference in the vitamin D status between the two major ethnic groups represented in this study. This may be expected because though different ethnic groups, the study participants were entirely black African race, with similar skin type (type IV–VI), and living under similar environmental and sociocultural conditions. Contrarily, the study in East Africa reported a significant association of vitamin D levels with ethnicity and proposed that it was probably due to different cultural and behavioural habits practiced by the different ethnic groups [16]. Most studies have also reported association of vitamin D levels with race, with the black race having the lowest levels of vitamin D [29, 30]. This is understood because of high melanin pigmentation that absorbs UV radiation, thus limiting the amount needed to convert 7-hydrocholesterol to vitamin D_3_ [1, 31].

This study also revealed that vitamin D status was not influenced by the number of hours spent outdoors and the percentage of body covered by clothing. Interestingly, those who covered their body less (<60%) had more hypovitaminosis D cases. It could be probably due to the climatic and cultural conditions that warrant more body covering for those who spent more time outdoors than when indoors. Also, the influence of time spent outdoors could have been obscured by the low prevalence of hypovitaminosis D in this study. Most of the participants did not own cars, thus they eventually at some point of the day had to walk out, either to take a cab or walk home, leading to some degree of outdoor exposure. In accordance with our study, Cabral et al. [22] found no significant association between sun exposure and vitamin D status among elderly men in Brazil. Contrarily, a study in the United Arab Emirates reported low vitamin D levels with increased Sun Avoidance Inventory score (which includes outdoor activities and dressing style), highlighting the effect of exposure to sun on vitamin D [25].

The community in which the participants belonged did not have any significant influence on the vitamin D level, indicating that the level or urbanization may not have had any effect on vitamin D in this study. These communities presented with different characteristics which was expected to have influenced their sun exposure habit. This is contrary to a cross-sectional study in China which revealed that those living in larger cities had higher levels of vitamin D inadequacy, and they proposed that it could be attributed to air pollution [32], which was not the case in any of the communities in this study. There was no significant difference of vitamin D levels or status with other indicators of socioeconomic status such as level of education, car ownership, income level, and marital status, though those with no income had the lowest levels of vitamin D. Those with no income were mostly the old who are most often more indoors and sedentary, with the added fact that vitamin D levels decreased with age and could have accounted for this observation. From these observations, socioeconomic status may not have had any influence on vitamin D status in this study. Bani-issa et al. [33] also observed higher prevalence of vitamin D deficiency among those with no job and the less educated which are indicators of low socioeconomic status. This is in contrast to results observed in Pakistan which revealed that lower levels of vitamin D were more associated with higher socioeconomic status [34]. We could not assess the effect of vitamin D supplementation on vitamin D status because, though some people indicated that they were on micronutrient supplements, many were ignorant of the composition of the supplement. This could have influenced to some extent the results obtained.

The first samples for vitamin D measurement were collected in July, August, and September which are considered as the peak periods of the rainy season in Cameroon, while the second samples were collected in December and January considered as dry season. The turnout of participant for the second sample collection was relatively much lower probably because the participants were no longer motivated since they already had their results of the first collection. There was no significant association with seasonal period of sample collection with vitamin D levels. It is expected that vitamin D levels should reduce in seasons when there is reduced or no sunshine as have been reported [35, 36]. Our results could probably due to the fact that even in the rainy season, there is still intermittent sunshine, leading to effective vitamin D production. Thus, it could be concluded that the sunlight intensity during the rainy season met the minimum threshold for vitamin D production. Also, the intense sunshine during the dry season could have enhanced shadow-seeking behaviour, leading to reduced exposure to direct sunlight. A study in India also observed no association between vitamin D and the different seasons [37]. Many studies have demonstrated a relationship of vitamin D with season. In China, vitamin D deficiency was more prevalent in spring than the other seasons [32]. Also Brock et al. [38] and Oleröd et al. [39] both observed that serum 25(OH)D levels were higher in summer and autumn than in winter and spring.

It could therefore be concluded that the prevalence of hypovitaminosis D is relatively low in this study population and of all the potential risk factors of vitamin D deficiency studied in this population, only age had a significant influence on vitamin D levels. This study involved apparently healthy individuals which could represent the healthy population of vitamin D status. Our future perspective would be to evaluate the vitamin D status in those with overt chronic diseases such as HIV, cancers, and tuberculosis in other to identify vitamin D deficiency risk groups for better healthcare intervention.

## Figures and Tables

**Table 1:** Comparison of median of vitamin D levels between groups of some risk factors.

Variable		*n*	25(OH)-vitamin D median (IQR) (mmol/L)	*p* value
Gender	Male	112	89.8 (23.7)	0.43
	Female	260	86.0 (27.4)	
Age group (years)	≤39	48	80.8 (23.9)	0.06
	40–49	90	91.9 (36.4)	
	50–59	113	90.1 (22.7)	
	60–69	75	83.8 (37.4)	
	≥70	46	82.1 (29.2)	
Ethnic origin	Bantu	136	84.9 (33.5)	0.25
	Semi-Bantu	228	88.8 (24.0)	
Community	Semiurban	126	85.0 (31.1)	0.12
	Semirural	93	80.9 (23.0)	
	Rural	45	87.2 (36.9)	
Educational level	Nil	81	81.9 (27.5)	0.54
	Primary	126	88.3 (26.0)	
	Secondary	86	89.4 (31.6)	
	Tertiary	77	91.2 (25.3)	
Income level	No income	52	79.9 (22.2)	0.09
	<1,00,000	210	89.3 (31.1)	
	1,00,000–2,00,000	41	84.8 (23.7)	
	>2,00,000	54	90.7 (25.2)	
Marital status	Unmarried	49	88.0 (29.1)	0.46
	Married	212	88.8 (25.2)	
	Divorced	8	94.0 (40.5)	
	Widowed	102	83.6 (29.2)	
Number of hours spent outdoors	<1 hour	44	82.9 (24.1)	0.22
	<3 hours	90	85.9 (33.2)	
	3–6 hours	128	89.6 (27.1)	
	6–10 hours	108	86.1 (28.6)	
% of body covered	>80%	72	87.2 (22.0)	0.50
	60–80%	284	87.2 (28.0)	
	<60%	14	82.6 (32.9)	

**Table 2:** Distribution of vitamin D status with some group variables.

Variable		*n*	Hypovitaminosis D, *n* (%)	Vitamin D sufficiency, *n* (%)	*p* value
Gender	Male	112	27 (24.1)	85 (79.5)	0.70
	Female	260	69 (26.5)	191 (73.5)	
Age group (years)	≤39	48	15 (31.2)	33 (68.8)	0.54
	40–49	90	23 (25.6)	67 (74.4)	
	50–59	113	22 (20.4)	90 (79.6)	
	60–69	75	21 (28.0)	54 (72.0)	
	≥70	46	14 (30.4)	32 (69.6)	
Ethnic origin	Bantu	136	41 (30.1)	95 (69.9)	0.12
	Semi-Bantu	228	52 (22.8)	176 (77.2)	
Community	Urban	126	38 (30.2)	88 (69.8)	0.91
	Semirural	93	27 (29.0)	66 (71.0)	
	Rural	45	12 (26.7)	33 (73.3)	
Educational level	Nil	81	22 (27.2)	59 (72.8)	0.89
	Primary	126	31 (24.6)	95 (75.4)	
	Secondary	86	24 (27.9)	62 (72.1)	
	Tertiary	77	18 (23.4)	59 (76.6)	
Income level	No income	52	15 (28.8)	37 (71.2)	0.72
	<1,00,000	210	55 (26.2)	155 (73.8)	
	1,00,000–2,00,000	41	12 (29.3)	29 (70.7)	
	>2,00,000	54	11 (20.4)	43 (79.6)	
Marital status	Unmarried	49	12 (24.5)	37 (75.5)	0.78
	Married	212	51 (24.1)	161 (75.9)	
	Divorced	8	2 (25.0)	6 (75.0)	
	Widowed	102	30 (29.4)	72 (70.6)	
Number of hours spent outdoors	<1 hour	44	12 (27.3)	32 (72.7)	0.21
	<3 hours	90	28 (31.1)	62 (68.9)	
	3–6 hours	128	25 (19.5)	103 (80.5)	
	6–10 hours	108	31 (28.7)	77 (71.3)	
% of body covered	>80%	72	12 (16.7)	60 (83.3)	0.06
	60–80%	284	78 (27.5)	206 (72.5)	
	<60%	14	6 (42.9)	8 (57.1)	

## Additional Points

*Limitations of Study*. Due to limited funding, it was not possible to measure 25(OH)D using the reference standard method which is LC-MS/MS. Parathyroid hormone could not be analysed because of technical aspects with respect to handling the samples to prevent the deterioration of the hormone. Also, another factor like body mass index which could influence vitamin D levels was not considered in this study. The participation for the second point sample collection was relatively low probable due to lack of motivation by the participants.

## Supplementary Materials

Supplementary Materials Research questionnaire: vitamin D status and its associated risk factors among adults in the Southwest region of Cameroon.
