# Supplementary material for: Vitamin D Status and Its Associated Risk Factors among Adults in the Southwest Region of Cameroon
Source: J Nutr Metab. 2018 Mar 19;2018:4742574. doi: 10.1155/2018/4742574 (PMC5884209; doi:10.1155/2018/4742574)
Supplement: Supplementary Materials — Research questionnaire: vitamin D status and its associated risk factors among adults in the Southwest region of Cameroon. [file 4742574.f1.pdf]

RESEARCH QUESTIONNAIRE

TITLE: **VITAMIN D STATUS AND ITS ASSOCIATED RISK FACTORS AMONG ADULTS IN THE SOUTHWEST REGION OF CAMEROON**

Name:______________________________________Tel. No._____________Code number: ______

Gender: Male ☐ Female ☐ Age (years): ______Religion: _____ Ethnic origin: _________

SOCIOECONOMIC STATUS

Educational level: ……………………………………………………|__|

[1) Nil 2) Primary 3) Secondary 4) Tertiary (Highest degree________________________)]

Employment status: ……………………………………………………|__|

a) [1) Unemployed 2) Employed]

b) Profession: ____

Monthly Income (Francs CFA): ……………………………………………|__|

[1) <100000 2)100000 – 200000 3) > 200000]

Residence:

1) Own a house: [1) Yes 2) No] ……………………………………………|__|

2) Renting: [1) Yes 2) No]…………………………………………………|__|

Do you own a car? [1) Yes 2) No] ………………………………………|__|

Marital status: [1) Unmarried 2) Married 3) Divorced 4) Widowed]……………|__|

If married is spouse employed? [1) Yes 2) No]……………………………|__|

Num of Children: ______________

LIFESTYLE ACTIVITIES

a) How many hours do you spend spent outdoors per day……………………|__|

1) <1hour 2) < 3 hours 3) 3-6 hours 4) 6-10 hours

b) What percentage of your body is covered by dressing per day……………|__|

1) > 80% 2) 60 – 80% 3) < 60%

c) Do you use sunscreen body lotions or creams? …………………………|__|

1) Yes 2) No

SMOKING STATUS

Do you smoke cigarettes? ………………………………………………|__|

[1) No 2) Occasional 3) Yes]

If yes, number of sticks per day: ……………………………………………|__|

[1) < 5 2) 5 – 10 3) 10 – 20 4) > 20]

Number of smoking years

Thanks for your cooperation
